# Altered functional connectivity of the nucleus accumbens subdivisions in amphetamine-type stimulant abusers: a resting-state fMRI study

**DOI:** 10.1186/s12868-019-0548-y

**Published:** 2019-12-30

**Authors:** Yun Wang, Kai-Juan Yan, Chen-Xiao Fan, Xiao-Nian Luo, Yuan Zhou

**Affiliations:** 1 The National Clinical Research Center for Mental Disorders & Beijing Key Laboratory of Mental Disorders, Beijing Anding Hospital, Beijing, China; 2 Advanced Innovation Center for Human Brain Protection, Capital Medical University, Beijing, China; 3 The Second Hospital of Jinhua City, Jinhua, Zhejiang China; 4 Key Laboratory of Behavioral Science & Magnetic Resonance Imaging Research Center, Institute of Psychology, Chinese Academy of Sciences, Beijing, China; 5 Department of Psychology, University of Chinese Academy of Sciences, Beijing, China

**Keywords:** Resting-state functional connectivity, Amphetamine-type stimulants abuse, Nucleus accumbens, Orbitofrontal cortex, Inferior frontal gyrus

## Abstract

**Background:**

The growing abuse of amphetamine-type stimulants leads to new challenges to human health. A possible addiction mechanism has been proposed by altered functional architecture of the nucleus accumbens (NAc) during resting state. NAc contains different subdivisions and they may play different roles in addiction. The aim of the present study was to examine whether there are common or distinct patterns of functional connectivity of the NAc subdivisions in amphetamine-type stimulant abusers (ATSAs).

**Methods:**

The present study recruited 17 male ATSAs and 22 healthy male controls. All the subjects underwent resting-state functional magnetic resonance imaging (fMRI) with their eyes closed. The NAc was divided into core-like and shell-like subdivisions. We used seed-based resting-state functional connectivity (RSFC) analyses to identify differences in brain functional architecture between ATSAs and healthy controls (HCs).

**Results:**

ATSAs had lower positive RSFCs with all of the NAc subdivisions over the left orbital part of superior frontal gyrus and higher positive RSFCs with the NAc subdivisions over the left opercular part of inferior frontal gyrus than HCs, which indicates common abnormalities across the NAc subdivisions in ATSAs. In addition, the RSFCs between the NAc subdivisions and the left orbital part of superior frontal gyrus were negatively correlated with the addiction severity in ATSAs.

**Conclusion:**

These results provide evidence that there are common RSFC patterns of the NAc subdivisions in ATSAs. The abnormality indicated by disrupted functional connectivity between the NAc subdivisions and prefrontal cortex suggests abnormal interaction between the rewarding process and cognitive control in ATSAs. Our results shed insight on the neurobiological mechanisms of ATSA and suggest potential novel therapeutic targets for treatment and intervention of ATSAs.

## Background

Drug addiction is one of the major health problems in the current society. The main feature of drug addiction is the inability to resist one's urge to obtain and take addictive drugs even though it can cause serious negative consequences [1]. Worldwide, the abuse of amphetamine-type stimulants is a global and growing phenomenon. In China, the proportion of amphetamine-type stimulants abusers (ATSAs) is continuously increasing over the past several years [2]. Drug addiction is often related to altered functional architecture in the brain, which results in hypersensitivity to the drug and drug-related cues and further ensures the compulsive drug-seeking behavior [3].

Recent neuroimaging studies have indicated the significant functions of the reward system in drug addiction. One of the main brain regions underlying addiction is the nucleus accumbens (NAc), a brain nucleus well recognized as a center of rewarding processes [4, 5]. Elevated dopamine transmission in the NAc is thought to be a primary mediator of drug addiction [6]. Besides, the NAc is a major input structure of the basal ganglia, thus it can integrate information from cortical and limbic regions and further modulate goal-directed behaviors. The NAc plays a pivotal role in refining action selection and mediating the rewarding effects of drugs abuse [7]. Therefore, it is generally thought that chronic exposure to addictive drugs disrupts the plasticity of the NAc, producing a pathologic motivation for addictive drug seeking.

Resting-state functional connectivity (RSFC) is shown to provide a measure of the brain’s functional organization [8]. Some researchers have studied the RSFC in substance-dependent populations, including individuals addicted to heroin [9–12], nicotine [13–16], and cocaine [17, 18]. These resting-state fMRI findings provide evidence that regions related to reward and cognitive control are involved in addiction [4]. A possible mechanism for drug addiction has been proposed by disrupted functional connectivity of the NAc in resting state [9]. It has been shown that the RSFC of NAc, especially its interaction with the prefrontal cortices, was related to impulsive behavior [19]. Exposures to addictive cues could decrease its RSFC to the prefrontal lobe [20, 21]. Further, the disrupted RSFC of NAc and prefrontal cortical regions has been reported in substance use disorder [22]. These findings demonstrate that drug addiction might be associated with altered functional connectivity between the NAc that involved in rewarding process and prefrontal cortical regions that involved in cognitive behavioral control processes.

Although functional connectivity of the NAc has been examined in drug addiction, no study has been conducted to investigate the functional connectivity of the NAc subdivisions. Numerous studies confirmed multi-aspect heterogeneity of the NAc, resulting the most intensely investigated shell-core dichotomy in animal models [23]. In human neuroimaging, multimodal connectivity-based parcellation also reveals a shell-core dichotomy of the human NAc [24]. The shell-like and core-like subdivisions of NAc were both found to facilitate the selection of the best reward functionally, but promote different patterns of behavior [7]. The shell-like subdivision, located in the ventromedial NAc, plays a role in the selection of the best reward by suppressing non- or less rewards stimuli that may obstruct with the best reward predicting stimuli. The core-like subdivision, located in the dorsolateral NAc, plays a role in the selection of the best reward by selectively stimulating incentive stimuli that are associated with the best reward [24]. All these researches indicate the dissociable roles of the NAc subdivisions to some extent. For now, no study has investigated the functional roles that different subdivisions of NAc may play in ATSA. Thus, it is not clear whether there are common changes of the RSFC across the different NAc subdivisions or distinct changes for each subdivision underlying the mechanism of addiction. The recently proposed data-driven NAc subdivision template in humans [24] makes it possible to evaluate the RSFC of each NAc subdivision in patients with ATSA, which will be important to shed light on the mechanisms of ATSA.

In brief, the RSFC of the NAc subdivisions in drug addiction has not been previously studied in addictive individuals. Therefore, this study aimed at investigating whether there are any addiction related alterations in RSFC of the NAc subdivisions by acquiring resting-state fMRI data from ATSAs and healthy controls (HCs). Previous studies emphasized the importance of the abnormal interactions between the NAc that process reward and prefrontal cortical regions that govern the cognitive-behavioral control in the mechanism of addiction, thus we hypothesized that the RSFCs between the NAc subdivisions and the prefrontal cortices were consistently altered among ATSAs. Meanwhile, considering the multi-aspect heterogeneity of the NAc, the regionally-dependent changes in RSFC of the NAc subdivisions may be revealed in ATSAs.

## Materials and methods

### Subjects

Male ATSAs (n = 17) were recruited from the Wuhan Mental Health Centre affiliated with the Huazhong University of Science and Technology during the period from October 2012 to December 2012. Healthy male controls (n = 22) were recruited from the local community and Huazhong University of Science and Technology by advertisements. The inclusion criteria for both groups were as follows: 18–40 years of age, male, at least 9 years of education, normal or corrected-to-normal hearing and vision, and no reported history of neurological problems, ophthalmic diseases, or severe head injuries. All enrolled ATSAs met the Diagnostic and Statistical Manual for Mental Disorders, 4th Edition (DSM-IV) criteria for drug dependence and were assessed using the Chinese version of the Addiction Severity Index (ASI-C) [25]. Clinicians assessed each participant’s addiction severity on seven areas: medical, employment/support status, alcohol, drug, legal, family/social, and psychiatric by using a 0–9 Likert scale [26]. A higher score indicates a more serious problem. The addiction severity of drugs was labeled as ASID. Furthermore, the enrolled ATSA participants all had used amphetamine-type stimulants for more than one year, and the accumulated dosage of the amphetamine-type stimulants they used was above 50 g. Based on self-reports of these abusers, among the 17 ATSAs, 8 participants used methamphetamine only and 9 participants used two or three amphetamine-type stimulants, such as methamphetamine, ecstasy or ketamine. None of the ATSAs and HCs had a history of abuse or dependence on other substances, with the exceptions of nicotine, caffeine, and alcohol. All of the ATSAs were inpatients, so they were in a state of withdrawal from any substance. They didn’t take any medicine or other treatment during hospitalization. One ATSA and one HC subject were later excluded from the study because of excessive head motion during the fMRI scan. This study was approved by the Ethics Committee of Wuhan Mental Health Centre. All participants or families of ATSAs provided written informed consent before participation.

### MRI data acquisition

Images were acquired with a 1.5 Tesla MRI scanner (Model: GE Signa HDxt) in Zhongshan Hospital, Wuhan City, Hubei Province, China. Whole-brain functional scans were collected using an echo-planar imaging (EPI) sequence (repetition time = 3000 ms; echo time = 40 ms; flip angle = 90°; matrix = 64 × 64; field of view = 220 × 220 mm^2^; number of slices = 33; slice thickness = 3 mm; slice gap = 1 mm). Each functional run contained 180 volumes. High-resolution T1-weighted images were acquired in a sagittal orientation employing a fast SPGR sequence (repetition/echo time = 9.176/2.956 ms; flip angle = 20°; slice thickness = 1.2 mm (no gap); number of slices = 128).

### Data preprocessing

Image preprocessing was performed using the Data Processing Assistant for Resting-State fMRI (DPARSF_v4.4, https://rfmri.org/DPARSF) [27], the Statistical Parametric Mapping (SPM12) program (https://www.fil.ion.ucl.ac.uk/spm), and the Resting-State fMRI Data Analysis Toolkit (REST 1.8, https://www.restfmri.net) [28]. Before preprocessing, we discarded the first 10 volumes to allow for signal stabilization. The remaining volumes were corrected for differences in slice acquisition times and then realigned to correct for small movements that occurred between scans. Subjects with a maximum displacement of more than 3 mm (in the x, y, or z direction) or more than 3° of angular rotation about any axis for any of the 170 volumes were excluded from the study. One ATSA and one HC were excluded from the analyses based on the recorded motion correction estimates. The realigned EPI images were coregistered to individual T1-weighted structural images. The locations of the NAc subdivisions in normalized T1 images for each subject can be seen in Additional file 1: Figure S1 and S2. Then the transformed structural images were segmented into gray matter, white matter, and cerebrospinal fluid [29]. Several sources of variance were removed from the realigned data by regression of nuisance variables, including 24 motion parameters (6 head motion parameters, 6 head motion parameters one time point before, and the 12 corresponding squared items), the signal averaged over the individual segmented cerebrospinal fluid and white matter (WM) regions, linear and quadratic trends [30]. The resulting maps were then registered into MNI space with 2 × 2 × 2 mm^3^ cubic voxels using the transformation information acquired from T1 image unified segmentation. A smoothing kernel of 4 mm was applied after registration. Finally, temporal filtering (0.01–0.1 Hz) of the time series was performed. To characterize differences in in-scanner microhead motion, the mean frame-wise displacement (FD), which includes measures of voxel-wise differences in motion in its derivation [31], was used as a measure of the micro-head motion of each subject [30].

### Definition of the regions of interest

**Fig. 1:**
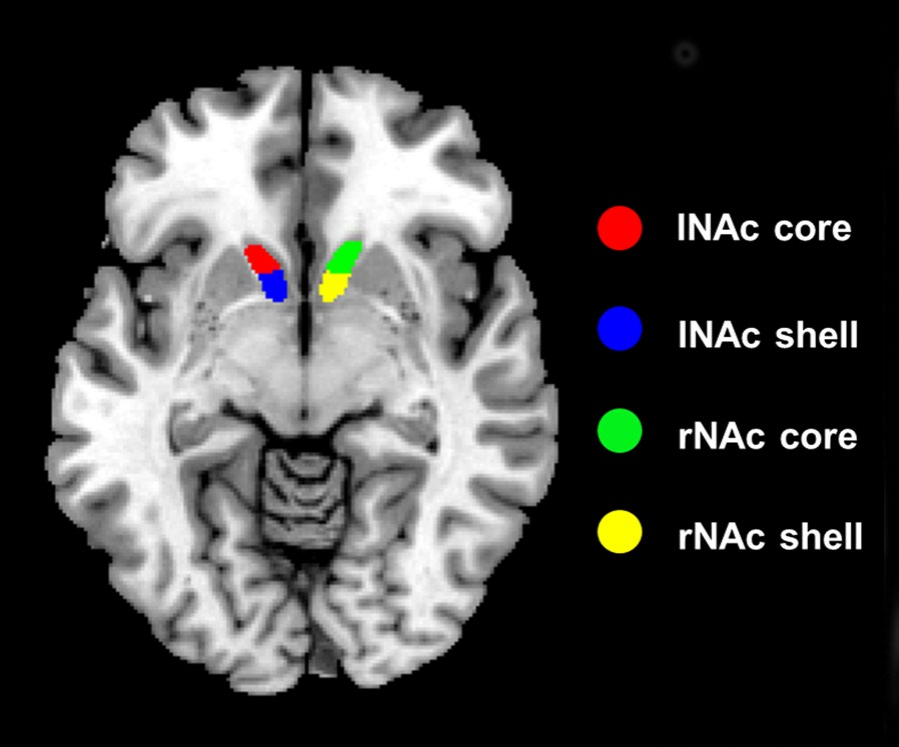
The four subdivisions of nucleus accumbens (NAc) intersected by Xia et al. [24]

Using neuroanatomy and histochemistry, the differentiation of the NAc subregions has been extensively studied, yielding a widely-accepted dichotomic shell/core-like subdivisions that reflect dissociable roles respectively. Recently, to investigate the regional differentiation within the NAc, Xia et al. [24] used three complementary parcellation schemes based on tractography, RSFC, and task-dependent co-activation and found that the 2-cluster solution with shell/core architecture provided the best description of the data. The clusters generated in this solution across the three parcellation schemes were defined as the final parcels [24]. In our study, the subdivisions of the NAc derived from RSFC patterns of these parcels were chosen as the regions of interests for further functional connectivity analyses (Fig. 1).

### RSFC analyses

The seed-based RSFC of the four subdivisions of the NAc was analyzed. By averaging the time series of all of the voxels within the seed region, the mean time series of each seed region was acquired. Pearson’s correlation coefficients were computed between the mean time series of the seed region and time series of each voxel of the whole brain. The correlation coefficients were converted into *z*-values using Fisher’s *r*-to-*z* transformation in order to improve their normality. The *z*-values were analyzed by one-sample *t*-tests to identify brain regions that exhibited significant positive or negative correlations with the seed region within each group (voxel-wise *p* < 0.001, cluster-wise FWE *p* < 0.05). Finally, the *z*-values were analyzed by two-sample *t*-tests to identify brain regions that exhibited significant differences in connectivity with the seed region between the ATSA group and HC group while accounting for the confounding effects of the education level, age and Jenkinson’s mean FD. The statistical threshold of the two-sample *t*-test was set at voxel-wise *p* < 0.001 in conjunction with a cluster-wise FWE *p* < 0.0125 to correct for multiple comparisons [*p* < 0.0125 was selected to take in account the number of independent seed regions used (0.0125 = 0.05/4)].

## Results

### Demographic data and head motion

The characteristics of the subjects and head motion information can be seen in Table 1. The mean ages of the two groups were not significantly different (*t* = 1.36, *p* = 0.18). The educational level of the ATSAs was significantly lower than that of the HC subjects (*χ*^*2*^ = 21.45, *p* < 0.001). The educational level and age were included as covariates in the following analyses. The mean FDs of the ATSA group and HC group were not significantly different (*t* = 1.49, *p* = 0.14). Considering the influence of head motion on intrinsic functional connectivity [32], we also included it as a covariate in the following analyses. The mean ASID score of ATSAs was 7.13.

**Table 1:** Demographic data and subject head motion

	HC	ATSA	*t/χ*^*2*^	*p*
Sample size	21	16		
Age (years)	29.52 ± 2.54	28.00 ± 4.24	1.36	0.18
Education			21.45	< 0.001
Junior high school	1	9		
Senior high school	0	2		
College degree	7	5		
Bachelor degree	13	0		
Mean FD	0.06 ± 0.03	0.05 ± 0.02	1.49	0.14
ASID		7.13 ± 1.93		

### RSFC analyses

**Fig. 2:**
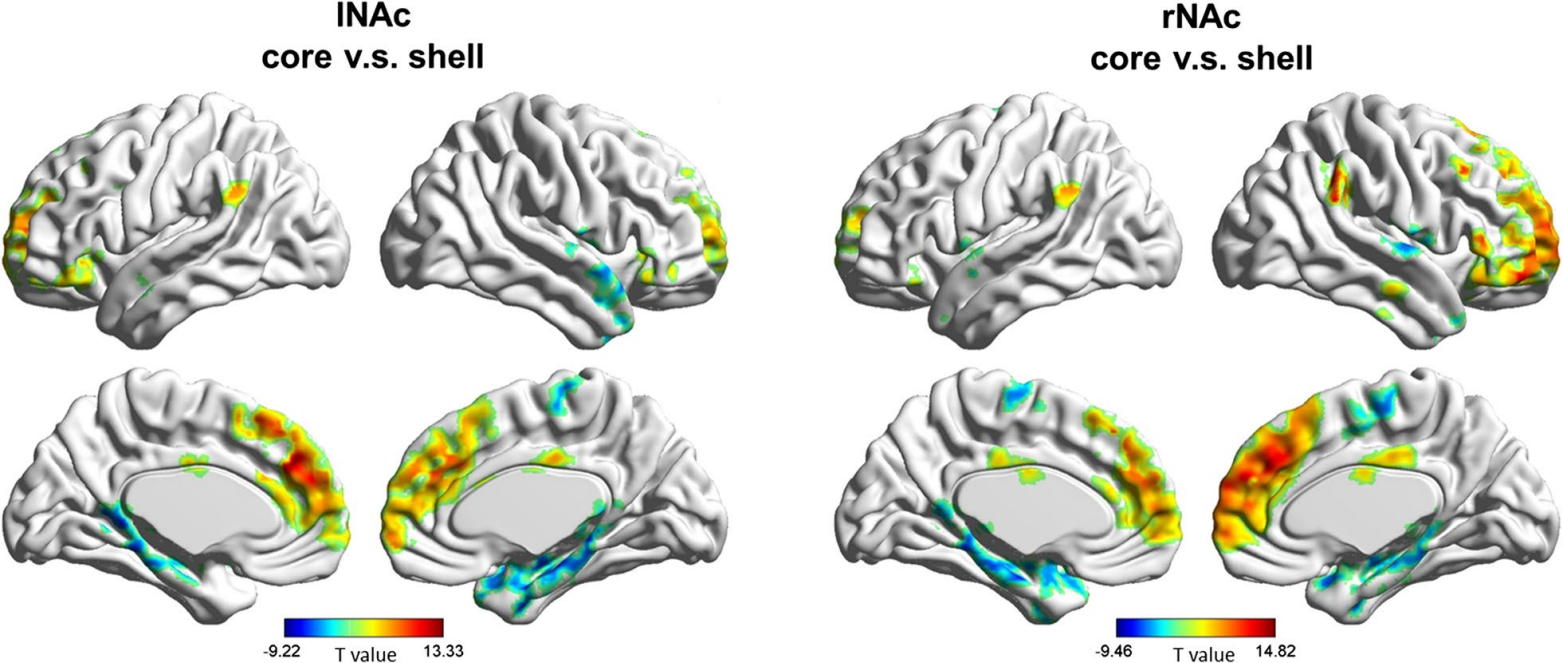
Regions showing significant differences in RSFCs with the left NAc core-like subdivision compared with the left NAc shell-like subdivision and significant changes in RSFCs with the right NAc core-like subdivision compared with the right NAc shell-like subdivision in the HC group. Warm colors represent the regions showing significant RSFCs with the NAc core-like subdivision compared with the NAc shell-like subdivision, and cool colors represent the regions showing significant RSFCs with the NAc shell-like subdivision compared with the NAc core-like subdivision. The images were created using BrainNet Viewer (https://www.nitrc.org/projects/bnv/)

In order to validate the parcellation of the NAc subdivisions, we used paired-sample *t*-test to examine whether there were any regions showing significant differences in RSFCs with the NAc core-like subdivision compared with the NAc shell-like subdivision in the HC group. We found that the positive RSFCs between the NAc core-like subdivision and the frontal gyrus, the cingulate gyrus and the inferior parietal lobule were significantly greater compared with the NAc shell-like subdivision and the positive RSFCs between the NAc core-like subdivision and the temporal gyrus, the parahippocampus gyrus, the insula, and the supplementary motor area were significantly smaller compared with the NAc shell-like subdivision in the HC group (cluster-level FWE *p* < 0.05; Fig. 2), which is consistent with the previous study [24].

**Fig. 3:**
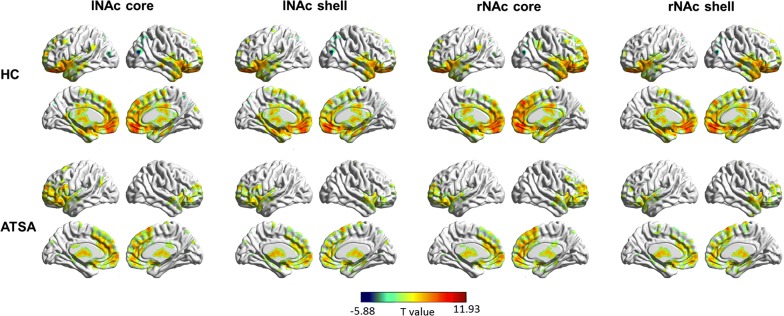
Spatial distributions of the RSFCs of the four seed regions within the HC group and the ATSA group. The spatial distribution of RSFCs was projected onto a surface brain using BrainNet Viewer (https://www.nitrc.org/projects/bnv/)

Then, we separately used each subdivision of the NAc as a seed region to reveal the specific networks that were influenced by amphetamine-type stimulant abuse. In general, the spatial distribution of the RSFC of each seed region in the HC group was larger than that of the ATSA group (Fig. 3). The brain areas that held positive RSFCs with the four NAc subdivisions were concentrated mainly in the insular, frontal gyrus, anterior cingulate cortex, orbitofrontal cortex, hippocampus, and parahippocampal gyrus in both the HC and ATSA group (cluster-level FWE *p* < 0.05). We also found negative RSFCs with all the NAc subdivisions in the precuneus, the superior parietal lobules and the inferior parietal lobules in the HC group (cluster-level FWE *p* < 0.05), while no significant negative RSFCs were found in the ATSA group. The connectivity pattern of each NAc subdivision in the HC group is similar to that in Xia’s study [24] by visual observations.

**Fig. 4:**
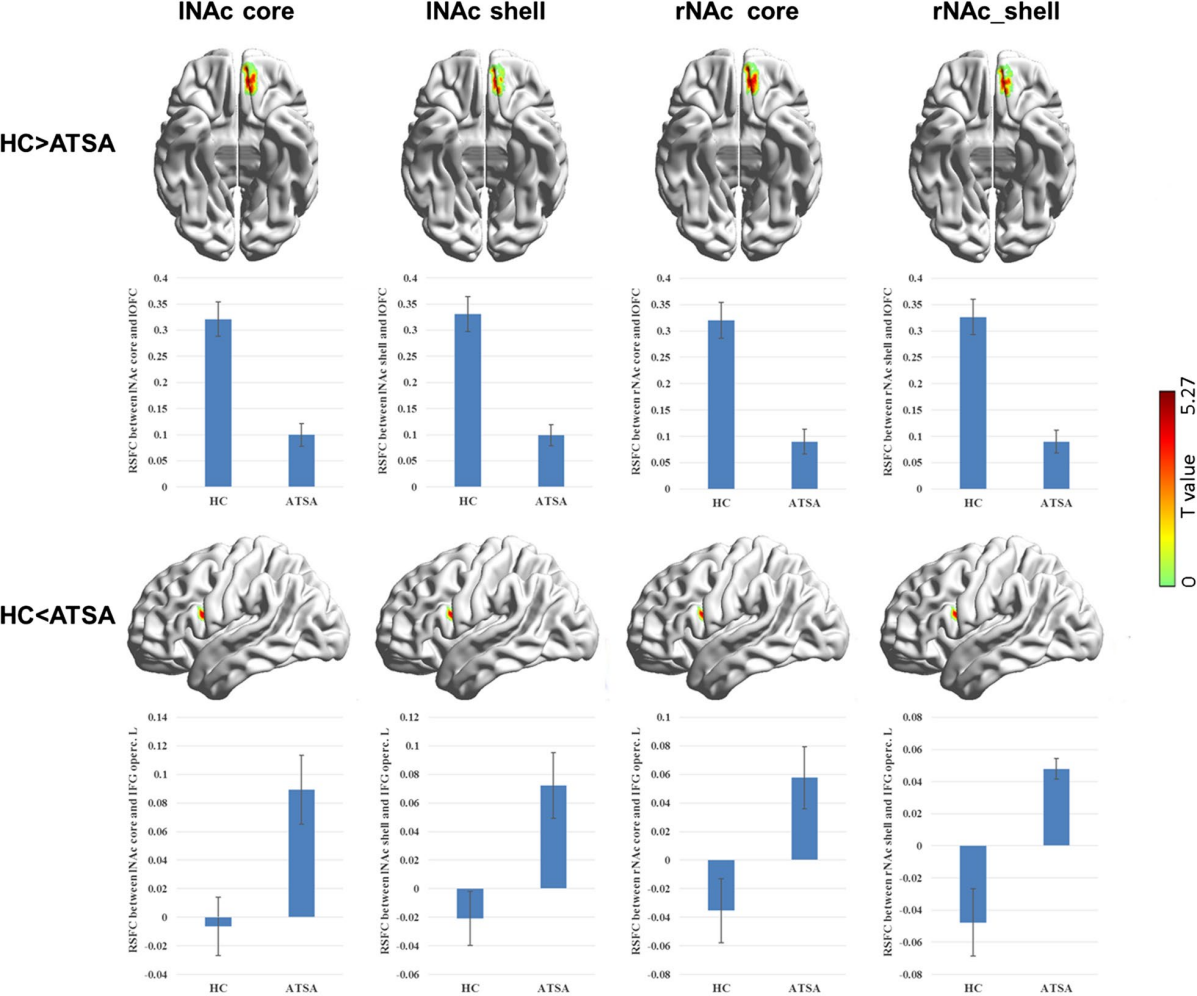
Regions showing significant changes in RSFCs with the seed regions in the ATSA group compared with the HC group and group comparisons of the connectivity strength in these two groups. The images were created using BrainNet Viewer (https://www.nitrc.org/projects/bnv/). Error bars indicate standard errors of the mean

By comparing the RSFC patterns between the two groups, functional connectivity for all the NAc subdivisions showed significant group differences (Table 2, Fig. 4). Specifically, in the ATSA group, all the four NAc subdivisions showed a decreased positive RSFC with the left orbital part of superior frontal gyrus (OFC) compared with the HC group (cluster-level FWE *p* < 0.0125). In addition, we found that the RSFCs between all the NAc subdivisions and the left opercular part of inferior frontal gyrus (IFGoperc) were increased in the ATSA group (cluster-level FWE *p* < 0.05 but *p* > 0.0125). We didn’t find any regionally-dependent changes in the RSFC of the NAc subdivisions. In addition, we also used masks to restrict our analyses of group comparisons in the voxels in which connections of the seed region were significant within the HC group or within the ATSA group. We found that the results were the same except for the RSFCs between the right NAc subdivisions and the left IFGoperc. That is, the increased RSFCs between the right NAc subdivisions and the left IFGoperc disappeared when applying the abovementioned mask.

**Table 2:** Group differences in functional connectivity between the ATSA group and the HC group

Seed region	Brain region	Hemisphere	BA	MNI coordinates	Peak T values	Cluster size	Cluster FWE *p* value
HC > ATSA							
lNAc core	Orbital part of superior frontal gyrus/orbital gyrus	Left	11/47	− 10 38 – 24	5.27	156	0.001
lNAc shell	Orbital part of superior frontal gyrus/orbital gyrus	Left	11/47	− 8 44 – 20	5.04	127	0.005
rNAc core	Orbital part of superior frontal gyrus/orbital gyrus	Left	11/47	− 10 44 – 22	5.27	145	0.002
rNAc shell	Orbital part of superior frontal gyrus/orbital gyrus	Left	11/47	− 8 44 – 20	5.21	122	0.007
HC < ATSA							
lNAc core	Opercular part of inferior frontal gyrus	Left	44	− 50 10 – 18	4.86	106	0.014*
lNAc shell	Opercular part of inferior frontal gyrus	Left	44	− 42 8 – 20	4.76	100	0.020*
rNAc core	Opercular part of inferior frontal gyrus	Left	44	− 46 4 – 18	4.72	91	0.032*
rNAc shell	Opercular part of inferior frontal gyrus	Left	44	− 42 12 – 16	4.84	97	0.023*

* These results can’t be corrected at the threshold of FWE *p* < 0.0125

**Fig. 5:**
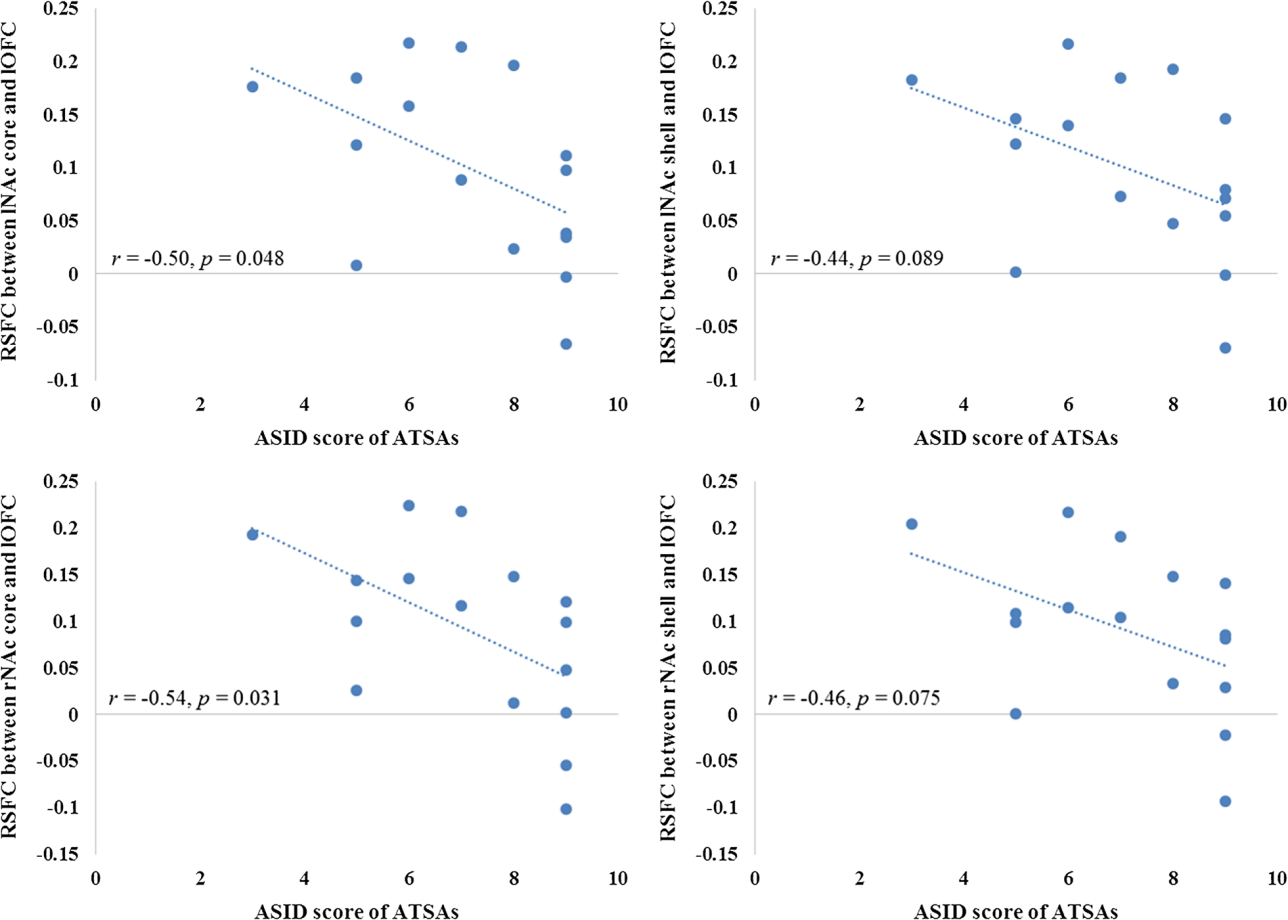
Correlations between addiction severity (measured by ASID) and the disrupted RSFCs of NAc subdivisions with the left OFC in the ATSA group

We further calculated the links between addiction severity (measured by ASID) and the strength of the abovementioned abnormal connectivity in the ATSA group. Results showed that the RSFCs between all the NAc subdivisions and the left OFC were negatively correlated with the ASID score in ATSAs (Fig. 5). The correlation coefficients were significant for the NAc core-like subdivisions (left: *r* = − 0.50, *p* = 0.048; right: *r* = − 0.54, *p* = 0.031) and marginally significant for the NAc shell-like subdivisions (left: *r* = − 0.44, *p* = 0.089; right: *r* = − 0.46, *p* = 0.075).

Given the commonalities of the NAc subdivisions in group-comparison results, we speculated whether the observed differences are due to general connectivity differences, not attributable to any specific subdivisions of the NAc. In order to test this possibility, we combined the NAc core-like and shell-like subdivisions in the same hemisphere as a seed region and conducted the RSFC analyses again. Results showed that no regions showed significant group differences in the connectivity of the left NAc. Only the left OFC (peak MNI coordinate: − 10, 46, − 24; cluster size: 89) showed significant group difference in the connectivity of the right NAc.

## Discussion

In the current study, we demonstrated that the RSFC of the NAc subdivisions was disrupted in ATSAs. Seed-based RSFC analyses revealed that the ATSA group showed altered RSFCs related to all the NAc subdivisions involved regions in the left OFC and the left IFGoperc. Besides, the RSFCs between the NAc subdivisions and the left OFC were negatively correlated with the addiction severity in ATSAs.

NAc is a central brain region of reward processing [4, 5]. It is well-accepted that most addictive drugs exert their initial reinforcing effects by inducing large dopamine rapidly in the NAc. This effect can render the drugs as highly salient, drive people’s strong motivations and stimulate compulsive drug-seeking behaviors [33, 34]. The OFC is a major brain region of cognitive impulse control. Previous study found that subjects with OFC lesions are more impulsive overall compared both to normal controls and to those with non-OFC brain damage [35]. By using transcranial direct current stimulation applied over OFC, researchers found participants had improved ability to inhibit inappropriate responses [36]. It is speculated that the elevated response in NAc combined with a disturbed cognitive control function in prefrontal cortex might lead to compulsive addictive behavior when facing rewarding cues [37]. Consistent with this speculation, reduced connectivity strength between the NAc and the OFC has been found in internet gaming disorder [38]. This suggests that abnormal interactions between the rewarding process and cognitive control could be associated with addictions. Our study showed that ATSAs had significantly reduced RSFC with the NAc subdivisions over the left OFC, supporting the disrupted connectivity between the NAc and prefrontal cortices involved in the mechanism of ATSAs. In addition, we found that the RSFCs between the NAc subdivisions and the left OFC were negatively correlated with the addiction severity in ATSAs. This showed that the more severe the ATSAs’ symptoms, the weaker the connectivity between the NAc subdivisions and the left OFC, which provided further evidence that the connectivity between the NAc and prefrontal cortices plays an important role in the pathophysiological basis of ATSAs.

We also found increased positive RSFCs between all the NAc subdivisions and the left IFGoperc when using FWE corrected *p* < 0.05 for multiple corrections. Many studies have addressed the role of the IFG in response inhibition [39–42]. Lesion studies in human and nonhuman primates have linked the IFG with the ability to inhibit inappropriate motor responses [42, 43] and functional neuroimaging studies also have implicated the IFG in response inhibition [44, 45]. In substance-dependent individuals, a previous study has found the association between years of cocaine use and go-nogo task-related brain activation. Specifically, greater activation in ventral striatum when facing cocaine cues and greater activation in the IFGoperc when facing response inhibition cues were found in subjects with more years of cocaine use [46]. In our study, there were no significant RSFCs between the NAc subdivisions and the left IFGoperc in the HC group while ATSAs had increased positive RSFCs between the left NAc subdivisions and the left IFGoperc. It should be noted that all the ATSAs recruited in the current study were inpatients and receiving detoxification during scanning. We speculated that these patients needed to constantly struggle with their addiction motivation during hospitalization. Thus, the increased RSFCs between the NAc subdivisions and the left IFGoperc in our study may suggest that ATSAs need to use effortful control resources to resist impulsive addictive drug-seeking behavior when they received detoxification. However, the finding of increased positive RSFCs between the NAc subdivisions and the left IFGoperc in ATSAs did not pass the strict threshold for multiple corrections (i.e., FWE corrected *p* < 0.0125), whether this connectivity is disrupted and how this connectivity plays a role should be further investigated in the future.

Our study has several limitations that should be noted. First, we only included males in the study, so the interpretation of the findings should be restricted to males. Secondly, the current findings should be replicated in future using images with higher spatial resolution. Thirdly, our sample size is small, future replications of this investigation are needed to verify these findings. Fourthly, three ATSA participants included in our study had a history of abuse or dependence on alcohol. Previous studies found that alcohol use was associated with weaker prefronto-striatal functional connectivity [47]. Abstinent alcohol-dependent adults also exhibited lower prefronto-striatal functional connectivity [48, 49]. Thus, the abnormal resting-state brain function observed in the ATSAs may be confounded by alcohol intake. ATSAs with history of abuse on alcohol may further reduce the RSFC between the NAc subdivisions and prefrontal cortex. Future studies with alcohol-naïve participants may distinguish the effects of alcohol on resting-state brain function from the effects of amphetamine-type stimulants per se. Lastly, we interpreted the results of RSFC based on the evidence of literature reviews. Thus, the specific functions of RSFC in the NAc subdivisions were not proven in our study. To assess the functions of these altered connectivities in ATSAs, well-designed studies are necessary in the future. For example, by combining fMRI with brain stimulation techniques such as transcranial magnetic stimulation on prefrontal cortex, we can observe the specific behavior changes in ATSAs and provide additional evidence for the role of the prefrontal-striatal circuit in ATSAs by moderating function of the prefrontal cortex involved in the prefrontal-striatal circuit. In addition, longitudinal studies across acute and remission periods might provide new insight into the characteristics of these alterations and determine whether these changes are trait or state markers in ATSAs.

## Conclusion

In summary, our result demonstrated that the NAc subdivisions, the center of reward processing [50], had altered RSFCs to prefrontal cortices among subjects with ATSA. These results provide evidence that there are common RSFC patterns of the NAc subdivisions in ATSAs. The common abnormality indicated by disrupted functional connectivity between the NAc subdivisions and the left OFC suggests abnormal interaction between the rewarding process and cognitive control in ATSAs. The abnormality of the RSFCs between the NAc subdivisions and the left IFGoperc may indicate that ATSAs need to use effortful control resources to self-regulate impulsive addictive behavior. Our results shed insight on the neurobiological mechanisms of ATSA and suggest potential novel therapeutic targets for treatment and intervention of ATSAs.

## Supplementary information

**Additional file 1: Figure S1.** Locations of the NAc subdivisions in normalized T1 images for each subject in the HC group.** Figure S2.** Locations of the NAc subdivisions in normalized T1 images for each subject in the ATSA group.

## Data Availability

The datasets used and/or analyzed during the current study are available from the corresponding author on reasonable request.

## Abbreviations

NAc: nucleus accumbens
ATSAs: amphetamine-type stimulant abusers
fMRI: functional magnetic resonance imaging
RSFC: resting-state functional connectivity
HCs: healthy controls
DSM-IV: Diagnostic and Statistical Manual for Mental Disorders, 4th Edition
ASI-C: Chinese version of the Addiction Severity Index
EPI: echo-planar imaging
WM: white matter
FD: frame-wise displacement
OFC: orbital part of superior frontal gyrus
IFGoperc: opercular part of inferior frontal gyrus
