# Supplementary material for: Altered functional connectivity of the nucleus accumbens subdivisions in amphetamine-type stimulant abusers: a resting-state fMRI study
Source: BMC Neurosci. 2019 Dec 30;20:66. doi: 10.1186/s12868-019-0548-y (PMC6937793; doi:10.1186/s12868-019-0548-y)
Supplement: Supplementary file 1 — Additional file 1: Figure S1. Locations of the NAc subdivisions in normalized T1 images for each subject in the HC group. Figure S2. Locations of the NAc subdivisions in normalized T1 images for each subject in the ATSA group. [file 12868_2019_548_MOESM1_ESM.docx]

## Additional file 1


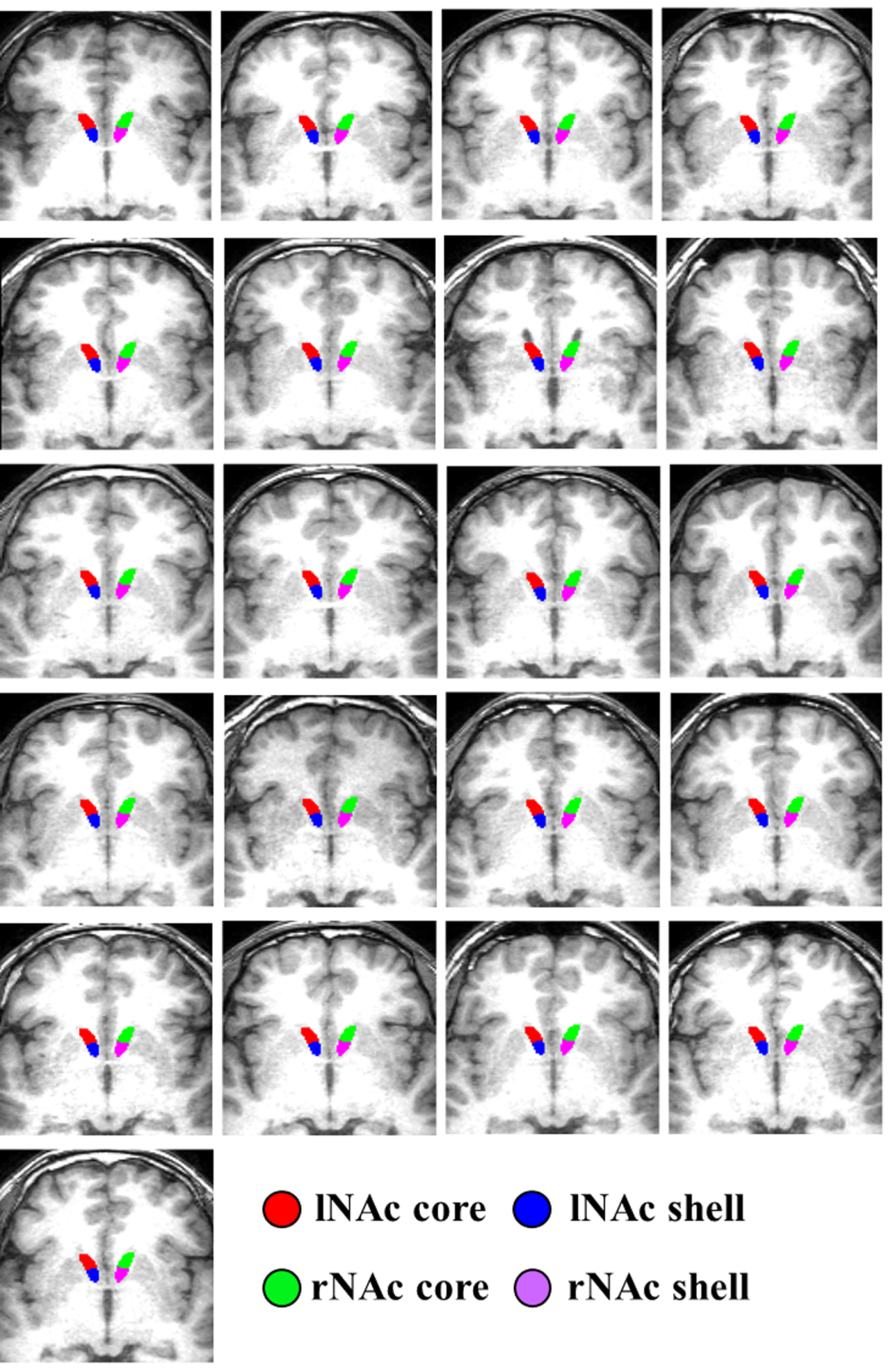


Figure S1 Locations of the NAc subdivisions in normalized T1 images for each subject in the HC group.


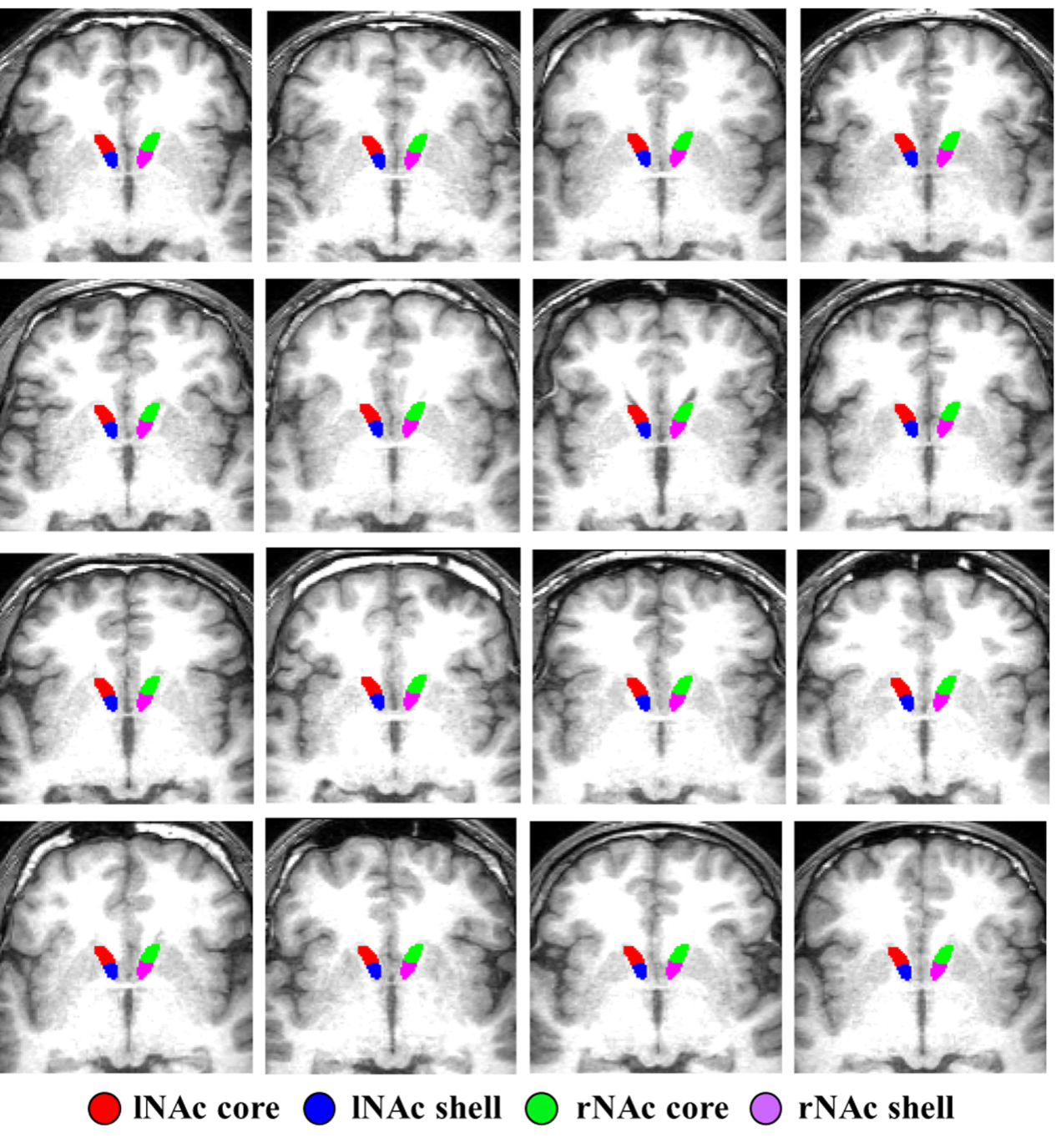


Figure S2 Locations of the NAc subdivisions in normalized T1 images for each subject in the ATSA group.
